# Comparative Study of Parenteral Penicillin G vs. Amoxicillin-Clavulanate for the Treatment of Dentoalveolar Abscess in Hospitalized Children

**DOI:** 10.3389/fped.2021.700188

**Published:** 2021-08-27

**Authors:** Amos Adler, Irit Gadot de-Vries, Jacob Amir, Liat Ashkenazi-Hoffnung

**Affiliations:** ^1^Clinical Microbiology, Tel-Aviv Sourasky Medical Center, Tel-Aviv, Israel; ^2^Sackler Faculty of Medicine, Tel-Aviv University, Tel-Aviv, Israel; ^3^Pharmacy Department, Schneider Children's Medical Center of Israel, Petach-Tikva, Israel; ^4^Department of Pediatrics, Mayanei Hayeshua Medical Center, Bnei Brak, Israel; ^5^Day Care Hospitalization Department, Schneider Children's Medical Center of Israel, Petach-Tikva, Israel

**Keywords:** dentoalveolar abscess, antimicrobial, parenteral therapy, hospitalized, surgical extraction

## Abstract

**Objectives:** To compare the clinical efficacy and the safety profiles of parenteral penicillin G vs. amoxicillin-clavulanate for the treatment of dentoalveolar abscess (DA) in hospitalized pediatric patients.

**Methods:** A retrospective cohort study that was conducted at the Schneider Children's Medical Center in Israel.

**Results:** Seventy-one patients that were included, 25 received parenteral penicillin G and 46 amoxicillin-clavulanate. There were no significant differences in the baseline clinical features except for higher rate of females in the amoxicillin-clavulanate group. Patients that were treated with penicillin G had shorter duration of fever, swelling and total length-of-stay (4.16 vs. 5 days in the penicillin G vs. amoxicillin-clavulanate groups, respectively, *p* = 0.007) and lower need for surgical intervention. Side effect were minor in both groups. In multivariate analysis, antimicrobial regimen was the only significant factor related with the total length-of-stay (*p* < 0.001).

**Conclusions:** In pediatric patients hospitalized for DA, parenteral penicillin G was associated with better outcome compared with amoxicillin-clavulanate.

## Introduction

Dentoalveolar abscess (DA) or odontogenic abscess are caused by infection of the dental pulp, secondary to untreated dental caries (1). Genera that are commonly found in these infections include the *Fusobacterium, Parvimonas, Prevotella, Porphyromonas, Streptococcus*, and the *Treponema* (2). Clinically, children typically present with facial swelling or cellulitis, and variably with fever or trismus. If not adequately treated, the infection might progress to life-threatening head and neck or systemic infections (2). Although milder cases can be managed with oral antimicrobials, severe cases are treated with parenteral antimicrobials and in some cases, surgical extraction of the infected tooth is necessary (3).

The use of antimicrobials for dental infections constitutes a significant share of antimicrobial use, even in northern Europe (4). However, the choice of antimicrobial regimen is not homogenous (1, 4), despite the overall high susceptibility of most oral pathogen to penicillin (2). This highlights the importance of comparative clinical data in order to determine the optimal choice of antimicrobial regimen, especially in the more severe, hospitalized patients.

The lack of uniformity in the choice of antimicrobial regimen are also present in our institution, where parenteral penicillin G (PCN) or amoxicillin-clavulanate (AMCL) are the most commonly used agents. Therefore, the goals of this study were to compare the clinical efficacy and the safety profiles of parenteral PCN vs. AMCL for the treatment of DA in hospitalized pediatric patients.

## Methods

This was a retrospective cohort study that was conducted at the Schneider Children's Medical Center, a tertiary, 270-bed pediatric hospital in Central Israel. Children admitted to one of the three General Pediatric (GP) wards were included if they met the following criteria: (1) age <18 year; (2) length of stay (LOS) > 24 hours; (3) parenteral treatment with either PCN or AMCL alone, at dosages of 37,000 IU/kg/dose QID for PCN or 25 mg/kg/dose TID for AMCL; (4) admission diagnosis of DA, determined by both a Pediatrician and a Maxillofacial surgeon. Exclusion criteria included (1) recent admission (<1 month) due to DA or (2) insufficient data in the medical records.

Data were collected from the patient's medical records and included: (1) demographic variables and comorbidities; (2) pre-admission illness and antimicrobial treatment; (3) clinical and laboratory characteristics at admission; (4) antimicrobial therapy and side effects; (5) outcome variables (duration of symptoms and hospitalization and the need for inpatient surgical extraction).

Statistical analysis was performed using the IBM SPSS statistic 25 version. Categorical variables were analyzed using χ^2^ test and continues variables were compared by the Mann-Whitney or the one-way ANOVA tests. Multivariate analysis was done by linear regression. A *p* < 0.05 was considered significant.

The study was approved by the Institutional Ethics Committee (No.: RMC-20-0166).

## Results

There were 118 patients admitted to the GP wards due to DA during the study period, of which 47 were excluded: 43 patients were treated with other antimicrobial combinations (>10 different regimens) and in four patients most of the data in the medical files was absent. Of the 71 patients that were included, 25 received parenteral PCN and 46 received AMCL. The average age was 7.4 years, and there were more female in the AMCL compared with the PCN group (57 vs. 28%, respectively, *p* = 0.03). There were no significant differences in the other baseline clinical or laboratory features upon admission (Table 1). Most patients (61%) received antimicrobial therapy prior to admission and almost all presented with swelling, albeit trismus was uncommon.

**Table 1:** Baseline characteristics of patients admitted with dentoalveolar abscess.

**Variable, n (%)^**a**^**	**All patients**	**Amoxicillin-clavulanate group**	**Penicillin group**	***P*-value**
Age (years), mean (SD)	7.4 (3.5)	7.4 (3.7)	7.2 (3.4)	0.8
Female gender	33 (46)	26 (57)	7 (28)	0.03
Arab Ethnicity (vs. Jewish)	15 (21)	11 (24)	4 (16)	0.76
Comorbidities^b^	12 (15.5)	8 (17)	3 (12)	0.74
Duration of symptoms prior to admission, mean (SD)	3.9 (4.2)	3.9 (3.8)	4.1 (4.9)	0.52
Admitting wardPediatric APediatric BPediatric C	25 (35) 24 (34) 22 (31)	19 (41)22 (48)11 (24)	6 (24) 2 (8) 7 (68)	<0.01
Pre-admission antibiotics:NoneAmoxicillinAmoxicillin-clavulanateBoth	29 (39) 15 (21) 17 (24) 11 (16)	9 (41)8 (17)11 (24)8 (17)	9 (36) 7 (28) 6 (24) 3 (8)	0.77
Body temperature at E.D. (°C), mean (SD)	37.6 (0.9)	37.6 (0.9)	37.5 (0.7)	0.42
Local swelling	70 (99)	45 (98)	25 (100)	NA
Trismus	9 (12.7)	6 (13)	3 (12)	0.9
Leukocyte count (10^3^ cells/mm^3^), mean (SD)	11.8 (3.5)	11.6 (3.5)	12 (3.6)	0.7
CRP^c^ (mg/dL), mean (SD)	4.9 (4.5)	5.3 (4.9)	4.1 (3.8)	0.42

a *for categorical variables;*

b *comorbidities included congenital heart disease (2), diabetes (4) and chronic granulomatous disease (1) in the Amoxicillin-clavulanate group, and rheumatologic disease (2), asthma (1) and cochlear implant (1) in the penicillin group;*

c *C-reactive protein*.

DA patients were randomly admitted to all three General Pediatric wards based on bed availability, but treatment with PCN was more common in ward C, compared with the other two (*p* < 0.01). Patients that were treated with PCN had better outcome in almost all measured variables (Table 2), including duration of fever and swelling, and the total LOS, that corresponded with the duration of parenteral antibiotics. The need for surgical intervention was also more common in the AMCL group (48 vs. 28% in the PCN group) but this difference was not statistically significant (*p* = 0.087). Culture were collected in only two cases, where “Viridans” type Streptococci and mixed gram-negative rods were identified. Only one case of abdominal discomfort was reported in a patient receiving AMCL and both groups had similar rates (~20%) of minor adverse events related to the peripheral intravenous access.

**Table 2:** Outcome variables of patients admitted with dentoalveolar abscess.

**Variable, n (%)^**a**^**	**All patients**	**Amoxicillin-clavulanate group**	**Penicillin group**	***P*-value**
Total length of stay^b^ (days), mean (SD)	4.7 (1.3)	5 (1.33)	4.16 (0.98)	0.007
Duration of fever (days), mean (SD)	0.8 (1.19)	1.09 (1.33)	0.28 (0.61)	0.012
Duration of swelling (days), mean (SD)	2.3 (0.94)	2.6 (0.93)	1.88 (0.73)	0.001
Duration of trismus (days), mean (SD)	0.3 (0.9)	0.39 (0.95)	0.28 (0.67)	0.68
Duration of parenteral fluids (days), mean (SD)	0.76 (0.9)	0.74 (0.98)	0.8 (0.87)	0.58
Duration of analgetics (days), mean (SD)	1.2 (1.37)	1.48 (1.44)	0.68 (1.1)	0.02
Surgical interventionNoneWithin 48 hAfter 48 h	42 (59) 12 (17) 17 (24)	24 (52)11 (24)11 (24)	18 (72) 1 (4) 6 (24)	0.087
Post-discharge antimicrobial treatment (days), mean (SD)	6.2 (2.4)	5.95 (2.5)	6.6 (2.3)	0.62

a *for categorical variables;*

b *corresponds with the duration of parenteral antimicrobial therapy*.

We evaluated the effect of the baseline characteristics on the duration of hospitalization which was the main outcome variable. In addition to the type of antimicrobial, other factors that correlated with the LOS (at *p* < 0.1) were the CRP value at the Emergency Department and the admission ward (data not shown). In multivariate analysis, the type of antimicrobial was the only factor that significantly correlated with the total LOS (β = 3.32, 95% C.I. 2.28–4.36, *p* < 0.001).

## Discussion

Although DA patients are typically treated initially by oral antimicrobials as outpatient, the majority of our patients had failed previous oral therapy or were considered severe enough to merit parenteral therapy. To the best of our knowledge, this study presents the first comparative data about the efficacy and safety of two parenteral antimicrobial regimens for the treatment of DA in hospitalized patients. Our results were surprising, as we did not expect to find that PCN therapy will be correlated with better outcome, as was manifested by both shorter duration of symptoms, parenteral antimicrobial and LOS and lower need for surgical intervention during hospitalization.

These results are in contradiction with previous comparative studies that reported faster recovery with oral AMCL (5) or even amoxicillin (6) compared with oral penicillin V, that has identical antimicrobial spectrum as PCN. Moreover, theoretically AMCL should have been superior to PCN thanks to its broader activity against certain anaerobes that can produce β-lactamase enzymes (2). The most likely explanation, beyond differences in study design and population are the differences in pharmacokinetic parameters between the oral and parenteral formulations. Whereas the oral formulation of AMCL has better oral absorption and serum levels compared with penicillin V (7), the dosage of parenteral PCN used in our study was ~30% higher in molar terms compared with AMCL (0.265 vs. 0.2 mMol/kg/day, respectively).

Microbiological studies from other countries showed that although no single antimicrobial is active against all species isolated from DA, the majority of these species are PCN-susceptible (2). Unlike other countries, DA cultures are rarely collected in Israel and thus no data exist regarding the susceptibility of DA isolates or even anaerobic bacteria in general in Israel. Nevertheless, as antimicrobial resistance in general is high in Israel (8), the results of our study provide reassurance regarding the microbiological and clinical adequacy of parenteral PCN for the treatment of DA.

There are several limitations in our study that are stemming from its retrospective nature, small sample size and the inclusion of one center only. Albeit these are valid points, we think that the study's results strongly support the conclusion regarding the favorable efficacy of parenteral PCN compared with amoxicillin-clavulanate, in pediatric patients hospitalized for DA, since this effect was manifest in several independent outcome measures and remained the only significant factor after multivariate analysis. This study also highlights the importance of pharmacokinetic factors as determinants of clinical efficacy. Hence, we believe that clinicians and researchers should not be deterred by the results of previous studies of oral PCN V (5, 6).

In the era of increasing antimicrobial resistance, the clinical use of “old” antimicrobials such as parenteral PCN is an important part of the battle against this trend. Thus, the use of parenteral PCN should be examined in future studies of DA and other head and neck infections.

## Data Availability Statement

The raw data supporting the conclusions of this article will be made available by the authors, without undue reservation.

## Ethics Statement

The studies involving human participants were reviewed and approved by the Ethics committee of the Schneider Children's Medical Center of Israel, Petach-Tikva, Israel. Written informed consent from the participants' legal guardian/next of kin was not required to participate in this study in accordance with the national legislation and the institutional requirements.

## Author Contributions

IG collected the data and performed the analysis. AA performed the analysis and wrote the manuscript. LA-H initiated the study and wrote the manuscript. JA assisted in designing the study. All authors contributed to the article and approved the submitted version.

## Conflict of Interest

The authors declare that the research was conducted in the absence of any commercial or financial relationships that could be construed as a potential conflict of interest.

## Publisher's Note

All claims expressed in this article are solely those of the authors and do not necessarily represent those of their affiliated organizations, or those of the publisher, the editors and the reviewers. Any product that may be evaluated in this article, or claim that may be made by its manufacturer, is not guaranteed or endorsed by the publisher.
